# Association of Adenotonsillectomy with Asthma Outcomes in Children: A Longitudinal Database Analysis

**DOI:** 10.1371/journal.pmed.1001753

**Published:** 2014-11-04

**Authors:** Rakesh Bhattacharjee, Beatrix H. Choi, David Gozal, Babak Mokhlesi

**Affiliations:** 1 Sections of Pediatric Pulmonology and Pediatric Sleep Medicine, Department of Pediatrics, University of Chicago, Chicago, Illinois, United States of America; 2 Center for Health and Social Sciences, University of Chicago, Chicago, Illinois, United States of America; 3 Section of Pulmonary and Critical Care, Department of Medicine, University of Chicago, Chicago, Illinois, United States of America; University of Manchester, United Kingdom

## Abstract

**Background:**

Childhood asthma and obstructive sleep apnea (OSA), both disorders of airway inflammation, were associated in recent observational studies. Although childhood OSA is effectively treated by adenotonsillectomy (AT), it remains unclear whether AT also improves childhood asthma. We hypothesized that AT, the first line of therapy for childhood OSA, would be associated with improved asthma outcomes and would reduce the usage of asthma therapies in children.

**Methods and Findings:**

Using the 2003–2010 MarketScan database, we identified 13,506 children with asthma in the United States who underwent AT. Asthma outcomes during 1 y preceding AT were compared to those during 1 y following AT. In addition, 27,012 age-, sex-, and geographically matched children with asthma without AT were included to examine asthma outcomes among children without known adenotonsillar tissue morbidity. Primary outcomes included the occurrence of a diagnostic code for acute asthma exacerbation (AAE) or acute status asthmaticus (ASA). Secondary outcomes included temporal changes in asthma medication prescriptions, the frequency of asthma-related emergency room visits (ARERs), and asthma-related hospitalizations (ARHs). Comparing the year following AT to the year prior, AT was associated with significant reductions in AAE (30.2%; 95% CI: 25.6%–34.3%; *p*<0.0001), ASA (37.9%; 95% CI: 29.2%–45.6%; *p*<0.0001), ARERs (25.6%; 95% CI: 16.9%–33.3%; *p*<0.0001), and ARHs (35.8%; 95% CI: 19.6%–48.7%; *p* = 0.02). Moreover, AT was associated with significant reductions in most asthma prescription refills, including bronchodilators (16.7%; 95% CI: 16.1%–17.3%; *p*<0.001), inhaled corticosteroids (21.5%; 95% CI: 20.7%–22.3%; *p*<0.001), leukotriene receptor antagonists (13.4%; 95% CI: 12.9%–14.0%; *p*<0.001), and systemic corticosteroids (23.7%; 95% CI: 20.9%–26.5%; *p*<0.001). In contrast, there were no significant reductions in these outcomes in children with asthma who did not undergo AT over an overlapping follow-up period. Limitations of the MarketScan database include lack of information on race and obesity status. Also, the MarketScan database does not include information on children with public health insurance (i.e., Medicaid) or uninsured children.

**Conclusions:**

In a very large sample of privately insured children, AT was associated with significant improvements in several asthma outcomes. Contingent on validation through prospectively designed clinical trials, this study supports the premise that detection and treatment of adenotonsillar tissue morbidity may serve as an important strategy for improving asthma control.

*Please see later in the article for the Editors' Summary*

## Abstract 2

Rakesh Bhattacharjee and colleagues use data from a US private health insurance database to compare asthma severity measures in children one year before and one year after they underwent adenotonsillectomy with asthma measures in those who did not undergo adenotonsillectomy.

*Please see later in the article for the Editors' Summary*

## Editors' Summary

**Background:**

The global burden of asthma has been rising steadily over the past few decades. Nowadays, about 200–300 million adults and children worldwide are affected by asthma, a chronic condition caused by inflammation of the airways (the tubes that carry air in and out of the lungs). Although asthma can develop at any age, it is often diagnosed in childhood—asthma is one of the commonest chronic diseases in children. In the US, for example, asthma affects around 7.1 million children under the age of 18 years and is the third leading cause of hospitalization of children under the age of 15 years. In people with asthma, the airways can react very strongly to allergens such as animal fur or to irritants such as cigarette smoke. Exercise, cold air, and infections can trigger asthma attacks, which can be fatal. The symptoms of asthma include wheezing, coughing, chest tightness, and shortness of breath. Asthma cannot be cured, but drugs can relieve its symptoms and prevent acute asthma attacks.

**Why Was This Study Done?:**

Recent studies have found an association between severe childhood asthma and obstructive sleep apnea (OSA). In OSA, airway inflammation promotes hypertrophy (excess growth) of the adenoids and the tonsils, immune system tissues in the upper airway. During sleep, the presence of hypertrophic adenotonsillar tissues predisposes the walls of the throat to collapse, which results in apnea—a brief interruption in breathing. People with OSA often snore loudly and frequently wake from deep sleep as they struggle to breathe. Childhood OSA, which affects 2%–3% of children, can be effectively treated by removal of the adenoids and tonsils (adenotonsillectomy). Given the association between childhood OSA and severe asthma and given the involvement of airway inflammation in both conditions, might adenotonsillectomy also improve childhood asthma? Here, the researchers analyze data from the MarketScan database, a large database of US patients with private health insurance, to investigate whether adenotonsillectomy is associated with improvements in asthma outcomes and with reductions in the use of asthma therapies in children.

**What Did the Researchers Do and Find?:**

The researchers used the database to identify 13,506 children with asthma who had undergone adenotonsillectomy and to obtain information about asthma outcomes among these children for the year before and the year after the operation. Because asthma severity tends to decrease with age, the researchers also used the database to identify 27,012 age-, sex-, and geographically matched children with asthma who did not have the operation so that they could examine asthma outcomes over an equivalent two-year period in the absence of complications related to adenotonsillar hypertrophy. Comparing the year after adenotonsillectomy with the year before the operation, adenotonsillectomy was associated with a 30% reduction in acute asthma exacerbations, a 37.9% reduction in acute status asthmaticus (an asthma attack that is unresponsive to the drugs usually used to treat attacks), a 25.6% reduction in asthma-related emergency room visits, and a 35.8% reduction in asthma-related hospitalizations. By contrast, among the control children, there was only a 2% reduction in acute asthma exacerbations and only a 7% reduction in acute status asthmaticus over an equivalent two-year period. Adenotonsillectomy was also associated with significant reductions (changes unlikely to have occurred by chance) in prescription refills for most types of drugs used to treat asthma, whereas there were no significant reductions in prescription refills among children with asthma who had not undergone adenotonsillectomy. The study was limited by the lack of measures of race and obesity, which are both associated with severity of asthma.

**What Do These Findings Mean?:**

These findings show that in a large sample of privately insured children in the US, adenotonsillectomy was associated with significant improvements in several asthma outcomes. These results do not show, however, that adenotonsillectomy caused a reduction in the severity of childhood asthma. It could be that the children who underwent adenotonsillectomy (but not those who did not have the operation) shared another unknown factor that led to improvements in their asthma over time. To prove a causal link, it will be necessary to undertake a randomized controlled trial in which the outcomes of groups of children with asthma who are chosen at random to undergo or not undergo adenotonsillectomy are compared. However, with the proviso that there are some risks associated with adenotonsillectomy, these findings suggest that the detection and treatment of adenotonsillar hypertrophy may help to improve asthma control in children.

**Additional Information:**

Please access these websites via the online version of this summary at http://dx.doi.org/10.1371/journal.pmed.1001753.

- The US Centers for Disease Control and Prevention provides information on asthma, including videos, games, and links to other resources for children with asthma
- The American Lung Association provides detailed information about asthma and a fact sheet on asthma in children; it also has information about obstructive sleep apnea
- The National Sleep Foundation provides information on snoring and obstructive sleep apnea in children
- The UK National Health Service Choices website provides information (including some personal stories) about asthma, about asthma in children, and about obstructive sleep apnea
- The “Global Asthma Report 2014” will be available in October 2014
- MedlinePlus provides links to further information on asthma, on asthma in children, on sleep apnea, and on tonsils and adenoids (in English and Spanish)

## Introduction

Asthma is a highly prevalent condition that currently ranks as the third most prevalent chronic disease in children, and is estimated to affect 7.1 million US children under the age of 18 y [1]. Furthermore, asthma is the third leading cause of hospitalization among US children under the age of 15 y [2], and in 2010 alone, there were approximately 640,000 pediatric emergency room visits related to asthma [3]. The consequences of asthma are further illustrated by the inordinately elevated annual direct health care costs of pediatric asthma, which amount to approximately US$50.1 billion in the US, with an additional indirect cost of US$5.9 billion attributable to lost productivity related to missed school days and, secondarily, missed work days for parents [4].

The mainstay of asthma control is adherent daily administration of anti-inflammatory therapies, including inhaled corticosteroids, inhaled corticosteroids combined with long-acting β_2_ agonists, and leukotriene receptor antagonists. Other important aspects aiming to optimize asthma control include avoidance of asthma triggers such as seasonal allergens and environmental pollution, particularly tobacco smoke exposure. However, recent studies have found asthma to be associated with several frequently overlapping co-morbidities including gastroesophageal reflux [5],[6], obesity [7]–[9], and sleep-disordered breathing [10]–[14], and have introduced plausible therapeutic strategies aimed at improving asthma control by minimizing or completely eradicating the burden of these coexisting conditions.

Obstructive sleep apnea (OSA) is considered the most prevalent and severe entity within the spectrum of sleep-disordered breathing, and affects approximately 2%–3% of all children [15]–[17]. Similar to asthma, the cardinal pathophysiological feature associated with childhood OSA involves the presence of increased airway inflammation, promoting the hypertrophy of upper airway adenotonsillar tissues [18]–[21]. The latter, either alone or in combination with other craniofacial and neuromuscular factors, promotes the presence of increased pharyngeal resistance. Thus, when the expected loss of the physiological dilator reflexes of the upper airway develops during sleep, these hypertrophic tissues and their underlying inflammatory components predispose the upper airway to episodically collapse, and cause the gas exchange abnormalities and fragmented sleep that characterize OSA [19]. Moreover, the repetitive upper airway obstruction during sleep and accompanying intermittent hypoxemia and hypercapnia, recurrent cortical microarousals, and sympathetic nervous system activation trigger oxidative stress and systemic inflammatory pathways that are currently believed to mediate OSA-associated morbidities [22]. Consequently, surgical adenotonsillectomy (AT) is considered the first line of therapy in childhood OSA and has recently shown overall favorable outcomes, including substantial improvements in the severity of respiratory sleep disturbances [23]–[25] and in reducing markers of OSA-associated systemic inflammation [26]. As anticipated, secular trends have revealed an increased frequency of AT in children over the past two decades related to a higher prevalence of obstructive breathing [27], although rarely is a formal diagnosis of OSA established through polysomnography by most otolaryngologists before AT [28].

Recent evidence has suggested that the pathophysiological complications of asthma and OSA may overlap in children, supporting the “united airway hypothesis” [29]. Numerous survey-based studies have reported a strong severity-dependent association between OSA and symptoms of asthma [10],[30]–[39]. Moreover, in a single-center cohort study of children with asthma, AT for those children who had polysomnographic evidence of OSA reduced the frequency of asthma exacerbations and of bronchodilator usage, and led to substantial improvements in pulmonary function [12]. However, to our knowledge, a systematic examination of the association of AT with asthma outcomes in a large sample of children has never been performed. To that end, we employed the MarketScan database, which contains a large longitudinal cohort of children with private health insurance, and extracted all those children with asthma diagnostic codes and asthma-specific pharmacy reimbursements. We examined the frequency of specific asthma outcomes before and after AT. Further, we compared these outcomes with matched children with asthma who did not undergo AT in order to evaluate the natural history of asthma over time [40]. We hypothesized that removal of the adenoids and tonsils, thereby improving sleep-disordered breathing and plausibly eradicating OSA, would be associated with substantial improvements in asthma control.

## Methods

### Ethics Statement

This study was approved by the University of Chicago's Institutional Review Board (BSD/UCH IRB approval no. 10-567-E).

### Data Source

All data were obtained using the MarketScan database, which provides access to a large database of over 180 million patients with private health insurance since 1995, including a large cohort of children (>25 million children). The MarketScan database collects payment information, capturing reimbursements from the health insurance plans and payments accrued by patients. It provides access to integrated patient-level data on expenses using billable codes in both the inpatient and outpatient settings, and also includes access to patient-level pharmacy expenses. Children were identified for a study period between January 1, 2003, and December 31, 2010.

### Participant Selection

Children aged 3 to 17 y with asthma were identified from the MarketScan database using asthma-specific International Classification of Diseases, Ninth Revision–Clinical Modification (ICD-9-CM) codes of asthma (493.XX). From this cohort, all children with asthma who had undergone AT were identified using current procedural terminology (CPT) codes specific for AT (CPT for AT for children aged <12 y is 42820, and for AT for children aged >12 y is 42821). Our cases (AT+) served as their own controls for the 1 y preceding AT. In addition, we studied a second group of matched children with asthma who did not undergo AT (AT−). These children were chosen if they met the above criteria for asthma, but did not have a history of any CPT codes for AT, adenoidectomy, or tonsillectomy. The purpose of adding this group was to include a cohort of children with asthma from the database in order to characterize the natural history of asthma when there is no confounding influence of complications related to adenotonsillar hypertrophy. This is of particular relevance since asthma severity improves with age, and recent evidence advocates the inclusion of a control group of children with asthma in studies examining the efficacy of an intervention in asthma [40]. AT− children were matched using a 2∶1 ratio to AT+ children, and were selected using a greedy selection algorithm according to age, sex, home location (urban or rural), and geographical state of residence based on a macro employed by SAS software, version 9.3. The greedy algorithm selects matched controls based on selecting the next ideal match in the fewest steps, without spanning the entire database to choose the best match.

**Figure 1:**
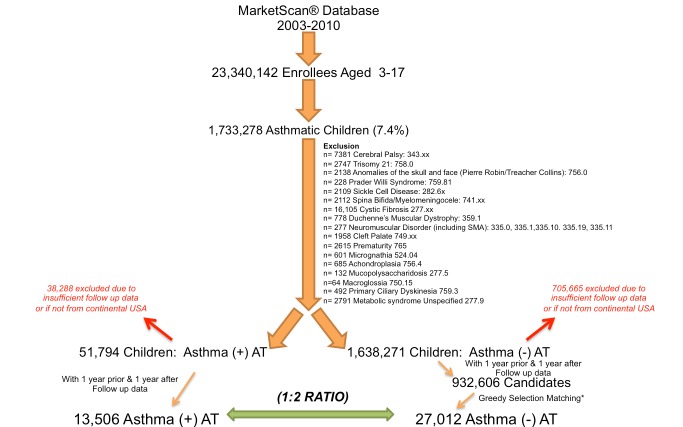
Selection of children from the MarketScan database following exclusion of specific comorbidities. Both AT+ and AT− children had to have data for 1 y prior to and 1 y after the index date (AT surgery or January 1) to be included. AT+ children were selected if they had an identified diagnostic code for asthma and AT. Matched AT− children were selected according to a 2∶1 ratio to AT+ children using a greedy selection algorithm. AT− children had a code for asthma without an identified diagnostic code for AT, adenoidectomy, or tonsillectomy. SMA, spinal muscular atrophy.

Because of the technical and ethical constraints of the MarketScan database, the dataset does not contain information on ethnicity or obesity status. In both AT+ and AT− children, the presence of ICD-9-CM codes corresponding to any confounding disease that could influence the respiratory tract and/or affect the efficacy of AT in the treatment of sleep-disordered breathing led to exclusion (Figure 1).

After implementation of the stringent selection criteria to form our study population, the temporal nature of study outcomes was entirely based on the timing of AT. For children with asthma who had undergone AT (AT+), the frequency of outcomes was examined during 1 y prior to the AT date and compared to during 1 y following the AT date. In children with asthma without a history of AT (AT−), the frequency of outcomes was examined over a similar 2-y period, such that AT− children had the same age, sex, home location, and geographical state as AT+ children at the time of AT, with the frequency of outcomes being defined as during the 1 y before and the 1 y after the arbitrary date of January 1. We examined a 2-y time period (1 y pre-AT and 1 y post-AT for AT+ children, and first and second year of follow-up for AT− children) in order to capture the frequency of asthma events over all four seasons. Of note, longer follow-up periods incurred a greater probability that children would drop out because of incomplete follow-up time or a higher likelihood that children would change health insurance providers. A clear limitation of the MarketScan database is that in the event that a child changes health insurance coverage, a new identification code is created, such that under these circumstances one child might be included as two separate children instead of one. Therefore, AT+ children without 1 y of pre-AT and 1 y of post-AT data and AT− children without 2 y of data under the same identification code were not included. Finally, only children from the continental United States and Alaska were included (i.e., children from Hawaii, Puerto Rico, Guam, and the US Virgin Islands were excluded).

### Study Outcomes

The primary outcomes for the present study were the frequency of ICD-9-CM codes (Table S1) for acute asthma exacerbation (AAE) and acute status asthmaticus (ASA). Secondary outcomes included the frequency of additional surrogates of asthma status such as ICD-9-CM codes (Table S1) for acute bronchospasm and wheezing, and CPT codes for spirometry, intubation, and initiation of “continuous inhalation for the first hour,” which would suggest an asthma exacerbation requiring continuous bronchodilator therapy, as is commonly performed in the emergency room setting.

In addition, since the MarketScan database provided outpatient pharmacy reimbursement claims in 10,663 AT+ children (79%) and 21,023 AT− children (78%), an additional secondary outcome was the frequency of asthma-specific outpatient medication prescription reimbursement (Table S2) during the same periods. For systemic corticosteroids we searched the database for generic and brand names of oral forms (tablet, syrup, elixir) of prednisone and prednisolone.

Finally, we examined all asthma-related emergency room visits (ARERs) and asthma-related hospitalizations (ARHs) by screening emergency room visits and hospitalizations in which the primary or secondary diagnostic code was billed for AAE or ASA (Table S1) or acute bronchospasm or wheezing (CPT codes).

### Statistical Analysis

Demographic data between AT+ and AT− children were compared using one-way ANOVA for age and chi-square for categorical outcomes. In the setting of multiple variables, such as in home location, a Wilcoxon matched-pairs signed rank test was used to compare AT+ to AT− children.

We used Fisher's exact test or chi-square with Yates' correction if the sample size was particularly large to compare the frequency of outcomes between AT+ and AT− children. Confidence intervals of proportions of a population were determined using a Wilson score interval.

## Results

For the study period January 1, 2003, to December 31, 2010, we identified 1,733,278 million children with asthma of a total of 23,340,142 million enrollees (ages 3–17 y) available in the database (prevalence of 7.4%) (Figure 1). Following exclusion of co-morbidities, we identified 51,794 children who had both an ICD-9-CM code for asthma and had undergone AT. Inclusion of children that had 1 y of data both pre- and post-AT reduced the number of AT+ children to 13,506 (Figure 1). Children excluded from the AT+ group because of insufficient follow-up data or because they did not reside in the continental United States did not markedly differ from children included in the AT+ group in distribution of age, gender, home location, and availability of pharmacy reimbursement claims. There were, however, observed differences in diagnostic codes provided for AT (Table S3). Following exclusion of co-morbidities, 932,606 candidate AT− children that fulfilled the 2-y follow-up requirement were identified from the database. Using a greedy selection algorithm with a target ratio of two AT− children for each AT+ child provided 27,012 AT− children who were successfully matched to AT+ children for age (Figure S1), sex, home location (Table 1), and geographical state of residence. Only the diagnostic codes of adenotonsillar hypertrophy, adenotonsillitis, sleep apnea, snoring, and sleep disturbance were significantly higher in the AT+ group (Table 1).

**Table 1:** Demographic summary of case (adenotonsillectomy) and control populations.

Characteristic	AT+ Group	AT− Group (2∶1 Match)	*p*-Value
**Number of children**	13,506	27,012	
**Diagnostic code**			
474.1× (hypertrophy of tonsils and adenoids)	5,942 (44%)	537 (2%)	<0.0001
474.0× (chronic tonsillitis and adenoiditis)	3,062 (23%)	137 (0.5%)	<0.0001
474.1×, 474.0× (hypertrophy of tonsils and adenoids and chronic tonsillitis and adenoiditis)	2,344 (17%)	265 (1%)	<0.0001
327.20, 327.23, 327.24, 327.26, 327.29, 780.51, 780.53, 780.57, 786.03, 780.50, 780.56, 780.59 (sleep apnea, snoring, and/or sleep disturbance)	3,603 (27%)	1,099 (1%)	<0.0001
**Age in years, mean ± standard deviation**	7.70±3.65	7.68±3.66	0.49
**Gender**			
Male	7,440 (55%)	14,880 (55%)	1.00
Female	6,066 (45%)	12,132 (45%)	
**With available pharmacy reimbursement claims**	10,663 (79%)	21,023 (78%)	1.00
**Home location**			0.98
**Metropolitan counties**			
Counties in metro areas of 1 million population or more	6,116 (45.2%)	12,249 (45.3%)	
Counties in metro areas of 250,000–1,000,000 population	2,746 (20.3%)	6,088 (22.5%)	
Counties in metro areas of fewer than 250,000 population	1,896 (14.0%)	3,597 (13.3%)	
**Nonmetropolitan counties**			
Urban population of 20,000 or more, adjacent to a metro area	756 (5.6%)	1,463 (5.4%)	
Urban population of 20,000 or more, not adjacent to a metro area	278 (2.1%)	607 (2.2%)	
Urban population of 2,500–19,999, adjacent to a metro area	1,024 (7.6%)	1,751 (6.5%)	
Urban population of 2,500–19,999, not adjacent to a metro area	442 (3.3%)	846 (3.1%)	
Completely rural or urban population of less than 2,500, adjacent to a metro area	124 (0.9%)	222 (0.8%)	
Completely rural or urban population less than 2,500, not adjacent to a metro area	124 (0.9%)	189 (0.7%)	

Values are number (percent) unless otherwise stated. *p*-Value is for the difference between the two groups.

**Figure 2:**
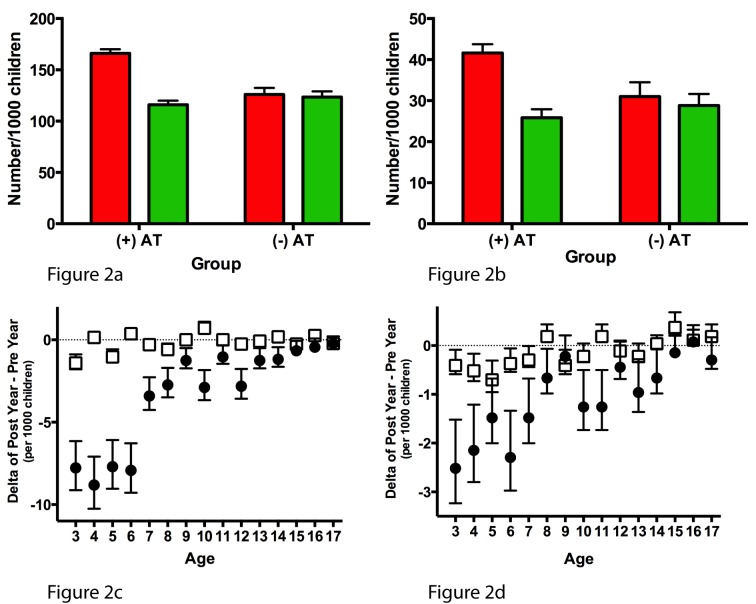
Annual incidence of primary asthma outcomes (acute asthma exacerbation and acute status asthmaticus). Annual incidence of AAE (A) and ASA (B) expressed per 1,000 children. Red bars represent 1 y prior to AT in AT+ children or the first year of follow-up in AT− children; green bars represent 1 y after AT or the second year of follow-up. The difference in annual incidence of AAE (C) and ASA (D) is calculated by subtracting the incidence during 1 y pre-AT (or first year of follow-up in AT− children) from the incidence during 1 y post-AT (or second year of follow-up in AT− children). Black circles represent the AT+ group, and white squares represent the AT− group. All error bars represent the 95th percentile confidence intervals for a sample proportion.

The frequency of episodes of AAE and ASA declined significantly in AT+ children following AT, while such frequency only slightly decreased in AT− children between the first year and the second year of follow-up. Indeed, the frequency of AAE was reduced from 2,243 pre-AT to 1,566 post-AT in the AT+ children, as compared to 3,403 in the first year and 3,336 in the second year in AT− children (30% versus 2% relative risk reduction; *p*<0.0001; Table 2). Similarly, the frequency of ASA decreased in AT+ children from 562 pre-AT to 349 post-AT, as compared to 837 in the first year and 778 in the second year in AT− children (38% versus 7% relative risk reduction; *p*<0.0001) (Table 2). When comparing each primary outcome individually, significant reductions associated with AT occurred in nearly all outcomes (Table S1). When the number of episodes was normalized per 1,000 children, the frequency of AAE and ASA was higher in children in the AT+ group prior to AT than in the AT− group in the first year of follow-up (Figure 2A and 2B). However, in the 1 y following AT, the frequency of AAE and ASA in AT+ children reached levels similar to those of AT− children in the second year of follow-up. The overall reductions in AAE and ASA (i.e., post-AT 1 y minus pre-AT 1 y) among AT+ children were present across the age spectrum (Figure 2C and 2D).

**Table 2:** Annual incidence of acute asthma exacerbation and acute status asthmaticus: comparing adenotonsillectomy to no adenotonsillectomy.

Item	AAE		ASA	
	AT+ Group	AT− Group	AT+ Group	AT− Group
1 y pre-AT/first year	2,243	3,403	562	837
1 y post-AT/second year	1,566	3,336	349	778
Percent reduction	30.0% (25.6% to 34.3%)	2.0% (−2.5% to 6.3%)	37.9% (29.2% to 45.6%)	6.8% (−2.6% to 15.4%)
*p*-Value		*p*<0.0001		*p*<0.0001

Data are number or percent (95% CI). *p*-Value is for the difference between the two groups.

**Figure 3:**
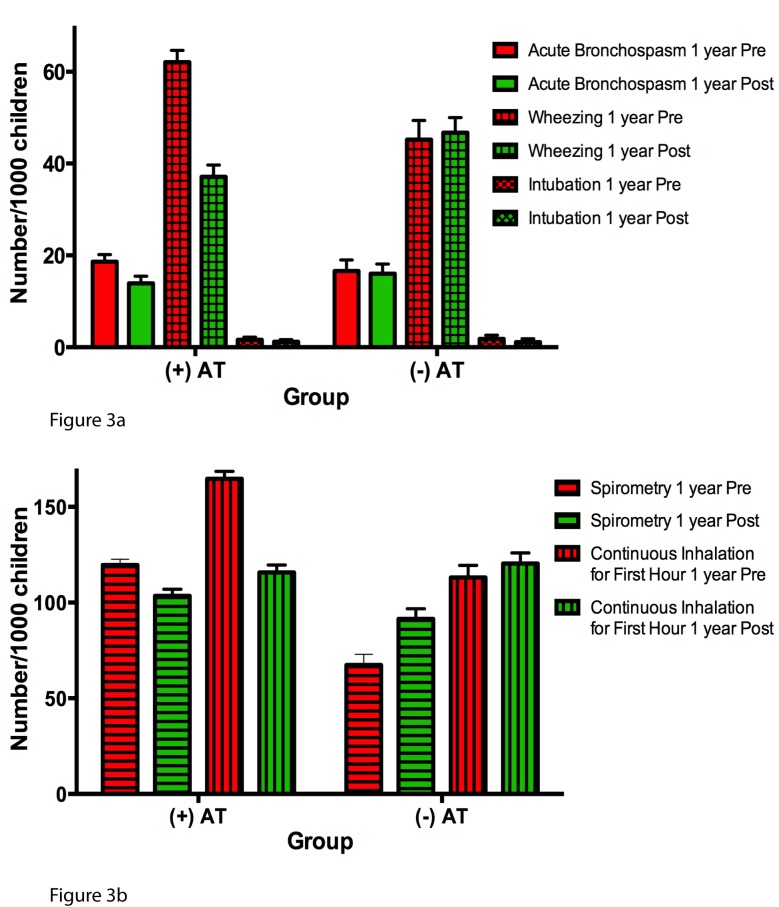
Annual incidence of secondary asthma outcomes expressed per 1,000 children. Red bars represent 1 y pre-AT in AT+ children or first year of follow-up in AT− children; green bars represent 1 y post-AT or second year of follow-up. All error bars represent the 95th percentile confidence intervals for a sample proportion. (A) Annual incidence of billing codes for acute bronchospasm, wheezing, and intubation. (B) Annual incidence of billing codes for spirometry and continuous inhalation for first hour.

Additionally, there were statistically significant reductions in health care provider coding for acute bronchospasm, wheezing, spirometry usage, and continuous inhalation therapy for the first hour in AT+ children following AT relative to corresponding matched AT− children in the analogous second year of follow-up (Table 3; Figure 3). The frequency of coding for endotracheal intubation, however, was not different between the two groups.

**Table 3:** Annual incidence of secondary asthma outcomes: comparing adenotonsillectomy to no adenotonsillectomy.

Item	Acute Bronchospasm		Wheezing		Spirometry		Continuous Inhalation for First Hour		Intubation	
	AT+ Group	AT− Group	AT+ Group	AT− Group	AT+ Group	AT− Group	AT+ Group	AT− Group	AT+ Group	AT− Group
1 y pre-AT/first year	251	448	839	1,222	1,615	1,820	2,225	3,054	22	49
1 y post-AT/second year	188	431	501	1,261	1,398	2,471	1,563	3,251	16	29
Percent reduction	25.1% (9.7% to 37.9%)	3.8% (−9.7% to 15.6%)	40.3% (33.5% to 46.4%)	0.0%	13.4% (7.4% to 19.1%)	0.0%	30.0% (25.4% to 33.8%)	0.0%	27.2% (−38.4% to 61.8%)	40.8% (6.4% to 62.6%)
*p*-Value		*p* = 0.04		*p*<0.0001		*p*<0.0001		*p*<0.0001		*p* = 1.00

Data are number or percent (95% CI). *p*-Value is for the difference between the two groups.

The information available on pharmacy reimbursements in the MarketScan database provides data regarding the number of prescription refills during the study period as well as the number of children filling a specific prescription. We chose to investigate both measures since in some children, particularly those with poorly controlled asthma, numerous prescriptions may have been refilled during the 2-y period. Regarding prescription refills, compared to AT− children, AT+ children having undergone AT had significant decreases in most classes of asthma prescriptions in the 1 y following AT. There was a 16.7% reduction in prescription refills for bronchodilators, a 21.5% reduction for inhaled corticosteroids, and a 13.4% reduction for leukotriene receptor antagonists (Table 4). However, there were no reductions in prescription refills of combination inhaled corticosteroids/long-acting β_2_ agonists, typically reserved for children with severe asthma, while in AT− children there was a 20.1% increase in the number of such prescriptions (Table 4).

**Table 4:** Annual prescriptions filled for asthma therapies: comparing adenotonsillectomy to no adenotonsillectomy.

Item	Bronchodilator		ICS		ICS/LABA		LTRA		Systemic Corticosteroids	
	AT+ Group	AT− Group	AT+ Group	AT− Group	AT+ Group	AT− Group	AT+ Group	AT− Group	AT+ Group	AT− Group
1 y pre-AT/first year	13,905	21,638	10,053	13,998	3,114	3,641	15,463	17,775	889	1,184
1 y post-AT/second year	11,580	21,070	7,887	14,276	3,182	4,373	13,386	18,852	678	1,098
Percent reduction	16.7% (16.1%–17.3%)	2.6% (2.4%–4.8%)	21.5% (20.7%–22.3%)	−2.0%	−2.2%	−20.1%	13.4% (12.9%–14.0%)	−6.1%	23.7% (20.9%–26.5%)	7.3% (5.8%–8.7%)
*p*-Value		*p*<0.0001		*p*<0.0001		*p*<0.0001		*p*<0.0001		*p* = 0.003

Data are number or percent (95% CI). *p*-Value is for the difference between the two groups.

ICS, inhaled corticosteroid; LABA, long-acting β_2_ agonist; LTRA, leukotriene receptor antagonist.

**Figure 4:**
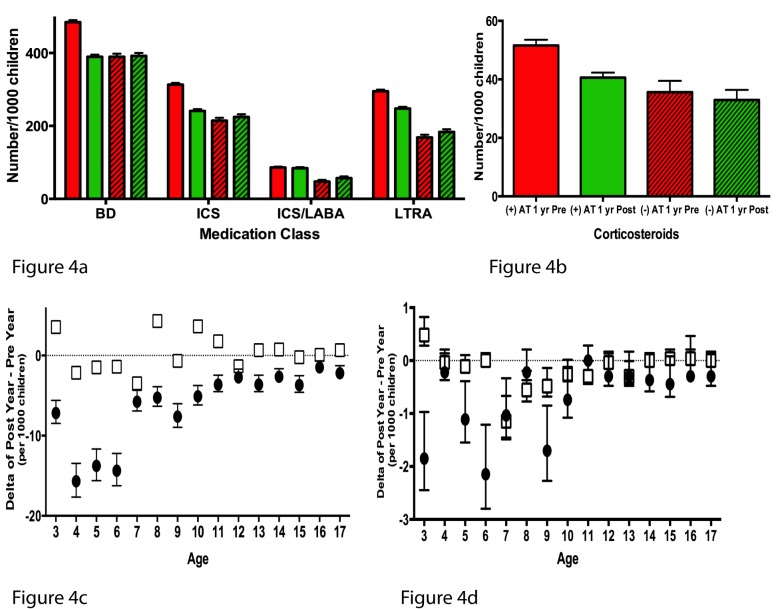
Annual number of children using asthma-specific prescription therapies. (A) Annual number of children using various asthma therapies expressed per 1,000 children. Solid red bars: AT+ group 1 y pre-AT; solid green bars: AT+ group 1 y post-AT. Red striped bars: AT− group first year of follow-up; green striped bars: AT− group second year of follow-up. (B) Annual number of children using systemic corticosteroids expressed per 1,000 children. (C) The difference in the number of children filling a prescription for a bronchodilator, subtracting the number during 1 y pre-AT (or first year of follow-up in AT− group) from the number during 1 y post-AT (or second year of follow-up). Black circles represent AT+ group means, and white squares represent AT− group means. (D) The difference in the number of children filling a prescription for a systemic corticosteroid therapy, subtracting the number during 1 y pre-AT (or first year of follow-up in AT− group) from the number during 1 y post-AT (or second year of follow-up). Black circles represent AT+ group, and white squares represent AT− group. All error bars represent the 95th percentile confidence intervals for a sample proportion. BD, bronchodilator; ICS, inhaled corticosteroid; ICS/LABA, inhaled corticosteroid with long-acting β_2_ agonist; LTRA, leukotriene receptor antagonist.

Upon examination of the number of children who filled various asthma prescriptions after normalizing per 1,000 children (Figure 4A), it is apparent that prior to AT, more AT+ children required prescriptions for bronchodilators, inhaled corticosteroids, and leukotriene receptor antagonists, and the number of AT+ children requiring these therapies declined to levels similar to those of AT− children following AT.

The number of prescription refills of systemic corticosteroids, which could indicate an asthma exacerbation, was markedly reduced in AT+ children following AT compared to the analogous second year of follow-up in AT− children (23.7% versus 7.3% reduction; *p* = 0.003) (Table 4). When examining the number of children needing systemic corticosteroids after normalizing the data per 1,000 children (Figure 4B), a larger proportion of AT+ children than AT− children needed systemic corticosteroids during the year prior to AT/first year of follow-up, but after AT, systemic corticosteroid use by AT+ children reached a level similar to that of AT− children in the second year of follow-up. Finally, assessment of the difference in the number of children filling a prescription for either bronchodilator or systemic corticosteroid therapies (Figure 4C and 4D, respectively) from the year pre-AT to the year post-AT revealed that the reduction of bronchodilator therapy post-AT was consistently present at all ages in AT+ children, while the reduction in systemic corticosteroids in AT+ children appeared to occur mostly in children <10 y of age.

**Figure 5:**
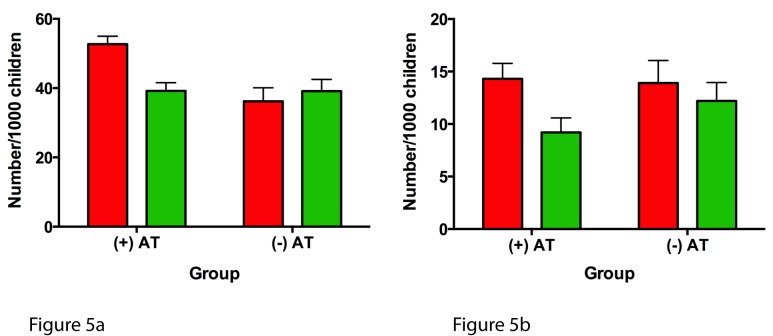
Annual frequency of asthma-related emergency room visits and asthma-related hospitalizations expressed per 1,000 children. (A) ARERs and (B) ARHs. Red bars represent 1 y prior to AT in the AT+ group and the first year of follow-up in the AT− group; green bars represent 1 y post-AT in the AT+ group and the second year of follow-up in the AT− group. All error bars represent the 95th percentile confidence intervals for a sample proportion.

The numbers of ARERs and ARHs were also significantly reduced following AT in AT+ children compared to AT− children in the second year of follow-up (25.6% versus 0.0% reduction; *p*<0.0001; and 35.8% versus 12.2% reduction; *p* = 0.0025) (Table 5). Following normalization of the number of children per 1,000 children (Figure 5), the frequency of severe asthma as related to the frequency of ARERs and ARHs was higher in AT+ children pre-AT and improved to levels comparable to those of AT− children following AT.

**Table 5:** Annual frequency of asthma-related emergency room visits and asthma-related hospitalizations: comparing adenotonsillectomy to no adenotonsillectomy.

Item	ARERs		ARHs	
	AT+ Group	AT− Group	AT+ Group	AT− Group
1 y pre-AT/first year	712	977	193	375
1 y post-AT/second year	530	1,056	124	329
Percent reduction	25.6% (16.9% to 33.3%)	0.0%	35.8% (19.6% to 48.7%)	12.3% (−1.6% to 24.3%)
*p*-Value		*p*<0.0001		*p* = 0.0247

Data are number or percent (95% CI). *p*-Value is for the difference between the two groups.

## Discussion

In this large sample of privately insured children with asthma, we examined longitudinal data and found that in children with asthma but without other significant comorbidities, AT was associated with significant decreases in asthma exacerbations as well as reductions in asthma-specific medication refills. The rationale of the current study was to examine whether the proposed overlap of two commonly inflammatory conditions in children, namely, asthma and OSA, leads to interdependent effects on the severity of the disease. To purposefully address our objective, we assumed that the primary indication for AT among most children in this cohort is for symptoms of OSA, and substantial evidence supports this assumption [27]. Accordingly, current findings support the possibility that the presence of OSA aggravates underlying asthma in children, and that treatment of OSA (via AT) might ameliorate the severity of asthma and reduce the need for more intensive use of anti-asthmatic medications. However, prospective trials are necessary to establish a causal relationship.

Before we discuss the potential implications of the current findings, some methodological issues deserve comment. We believe that the use of AT CPT codes provides a reasonably reliable surrogate indicator for the presence of OSA, particularly considering that during the period covered in the study, the vast majority of ATs were performed as treatment for OSA [27]. We deliberately used CPT codes for AT instead of ICD-9-CM codes for OSA. A priori evaluation of our AT+ and AT− children revealed that only 842 (6.2%) of the AT+ children and 159 (0.6%) of the AT− children had undergone some form of evaluation for sleep-disordered breathing as determined by searching for specific sleep diagnostic testing CPT codes (Table S4). This observation is consistent with previous studies indicating that the vast majority of children undergoing AT are unlikely to undergo formal diagnostic polysomnography testing for OSA [41],[42]. In order to capture the large population of children with OSA treated with AT in whom diagnostic testing was not conducted, confirmation of OSA by concurrent utilization of the sleep diagnostic CPT codes was precluded. Given the secular trends whereby OSA is the major indication for AT in up to 80% of children [27], we surmise that most children in our study underwent AT for symptoms of OSA. Despite the paucity of diagnostic polysomnography in the AT+ group, 27% of children did have an insurance claim for a diagnostic code of sleep apnea, snoring, or sleep disturbance, and 44% of children had an insurance claim for adenotonsillar hypertrophy during the 1-y period prior to surgery. Only 23% of children had an isolated insurance claim of chronic tonsillitis and adenoiditis (Table 1).

In addition, the diagnosis of asthma is particularly problematic in younger children, given their limited ability to participate in diagnostic pulmonary function testing including methacholine or exercise bronchial provocation testing. Moreover, virally induced wheezing during early childhood may often be incorrectly labeled as asthma. To partially overcome this limitation, we separately analyzed children under 6 y of age and older children (Table 6). The associations between AT and improvements in asthma outcomes remained essentially unchanged from analyses in which all children were included independent of age.

**Table 6:** Annual incidence of acute asthma exacerbation and acute status asthmaticus in children <6 and children ≥6 y: comparing adenotonsillectomy to no adenotonsillectomy.

Item	AAE		ASA	
	AT+ Group	AT− Group	AT+ Group	AT− Group
**Ages 6–17 y**				
1 y pre-AT/first year	1,286	2,111	343	490
1 y post-AT/second year	937	2,106	213	475
Percent reduction	27.1% (21.0% to 32.8%)	0.2% (−5.7% to 5.9%)	37.9% (26.4% to 47.6%)	3.1% (−9.9% to 14.5%)
*p*-Value		*p*<0.0001		*p*<0.0001
**Ages 3–5 y**				
1 y pre-AT/first year	957	1,292	219	347
1 y post-AT/second year	629	1,230	136	303
Percent reduction	34.3% (27.5% to 40.4%)	4.8% (−2.7% to 11.8%)	38.2% (29.2% to 46.0%)	12.7% (−1.8% to 25.1%)
*p*-Value		*p*<0.0001		*p* = 0.0115

Data are number or percent (95% CI). *p*-Value is for the difference between the two groups.

Notwithstanding the challenges of identifying correctly the diagnosis of OSA and asthma using the MarketScan database, it is well established that the similarities between asthma and OSA in children are quite extensive. Indeed, both are considered inflammatory disorders of the airways as well as low-grade systemic inflammatory diseases [20],[43],[44]. Asthma and OSA also share risk factors such as allergic rhinitis, obesity, exposure to tobacco smoke, frequent respiratory infections, and African-American race [45],[46]. In addition, rhinitis, commonly associated with OSA, is also considered a precursor to asthma [47]. The association of allergic rhinitis with both OSA and asthma suggests biological plausibility for the idea that OSA is related to asthma, and suggests that in the treatment of OSA, AT potentially improves asthma status by reducing the burden of allergic disease and inflammatory disease of the upper airway. Moreover, the seasonal variation of pediatric OSA is remarkably similar to the seasonal variation of asthma status [48]–[50]. Thus, although this study supports an association and by no means confirms causality, there is a rationale to support a biologically plausible link between asthma and adenotonsillar morbidity, namely OSA.

Furthermore, a relatively large number of questionnaire-based studies assessing symptoms of asthma or the presence of snoring have thus far supported the presence of an association between OSA and recurrent wheezing and/or asthma across many regions and states [10],[30]–[39]. Notwithstanding the limitations of such questionnaire-based surveys, there appears to be a severity-dependent effect, whereby more severe OSA is associated with poorer asthma control [11]. Although randomized controlled studies are needed to prove cause and effect between OSA and asthma, the present findings provide a compelling argument in favor of such studies, considering the putative improvements that emerged in asthma-related health care utilization following AT.

The current results are supportive of the findings in three previous studies [12],[51],[52]. Saito and colleagues [51] reported that in 25 patients with asthma, AT improved asthma symptoms in 88%, with 60% of children able to eliminate all of their asthma therapies, and 28% able to eliminate some of their therapies [51]. In a retrospective study of 93 children with asthma, AT was associated with significant reductions of asthma therapies, including corticosteroids, and with improvements in asthma [52]. Finally, Kheirandish-Gozal and colleagues [12] demonstrated in a prospective study that AT for polysomnographically demonstrated OSA was associated with reduced symptoms and rescue inhaler therapy usage in 35 children with poorly controlled asthma, and further reported significant improvements in pulmonary function following AT. However, these three studies included small sample sizes and short follow-up periods that clearly limit their generalizability [12],[51],[52]. None of these studies compared children with asthma who underwent AT to a group of children with asthma who did not undergo AT, an important issue since improvements in asthma status seen following AT in these three studies could also be related to seasonal variation, or related to a spontaneous improvement in asthma control over time through growing older [40]. In order to address these limitations, our study included a very large sample of privately insured children with asthma who underwent AT compared to children with asthma who did not undergo AT. Moreover, we took advantage of the MarketScan database, which includes features of health care resource utilization over time for our longitudinal comparisons. Although the database provides information on a large sample of privately insured children, it does not include any data on the large group of uninsured children or those receiving health care services through public health insurance such as Medicaid. On the other hand, our data exclude the confounding effect of lack of access to health care, as all children included in our cohort were privately insured.

Our study has several additional limitations that are primarily inherent to the analysis of large administrative databases. Some of our outcomes were based entirely on physician billing practices. While there is a possibility of coding error or reporting bias, there is no reason to assume that such errors or biases differ among AT+ and AT− children. Furthermore, the inclusion of data on prescription refills as an additional outcome measure in this study provides additional objective outcomes that are not dependent on physician billing practices. As mentioned earlier, the MarketScan database does not provide information regarding the ethnicity of children. The racial disparities regarding health care utilization are very relevant to asthma control [53],[54]. In addition, data were not available for obesity status, and therefore we were unable to control for body mass index, another important risk factor in asthma prevalence and severity [7],[55]. We acknowledge that race and obesity are important confounding variables for asthma status, and the unavailability of data on both race and obesity status represents an important limitation, particularly with regard to our selection of matching control children with asthma who did not undergo AT. In spite of this limitation, we believe that matching a child's home location and geographical state to the level of county of residence—a surrogate of a child's socioeconomic status—can serve to minimize any potential discrepancies in race and obesity status between the two groups (Table 1) [56]. Although our dataset included children who received services up to 2010, we do not anticipate any change in the association between AT and asthma since 2010.

Notwithstanding the study's limitations, using a large sample size of carefully selected children with asthma who underwent AT, we demonstrated that AT was associated with reduced asthma severity and improved control. Moreover, asthma-related outcomes in children after AT were comparable to those in children with asthma who did not undergo AT. While recent evidence reveals that the most common reason for AT in the United States is OSA [27], it is important to acknowledge that this commonly performed surgery is not without risk. Morbidities can range from relatively minor hemorrhage and dehydration to more severe complications including anoxic brain injury and death [57],[58].

In summary, our findings provide additional evidence that AT is associated with improved asthma control and suggest that OSA is also associated with the severity of asthma in these children. Further research is needed to better elucidate the pathways linking OSA with asthma in order to establish biological plausibility and, finally, to establish the criteria for identification of those children with asthma most likely to develop OSA and to potentially benefit from AT.

## Supporting Information

Checklist S1 STROBE research checklist.
(DOCX)

Figure S1 Age distribution of the AT+ and AT− groups. Matched AT− group children were chosen using a 2∶1 ratio; hence, at each age point there is an anticipated doubling in the AT− group (green bars) for the corresponding AT+ group (red bars).
(TIF)

Table S1 ICD-9-CM and CPT codes used as study outcomes.
(DOCX)

Table S2 Asthma pharmacy classes used as study outcomes.
(DOCX)

Table S3 Demographic summary of children included in and excluded from the AT+ group.
(DOCX)

Table S4 CPT codes used for sleep apnea diagnostic testing.
(DOCX)

## Abbreviations

AAE: acute asthma exacerbation
ARER: asthma-related emergency room visit
ARH: asthma-related hospitalization
ASA: acute status asthmaticus
AT: adenotonsillectomy
CPT: current procedural terminology
ICD-9-CM: International Classification of Diseases, Ninth Revision–Clinical Modification
OSA: obstructive sleep apnea
