# Supplementary material for: Association of Adenotonsillectomy with Asthma Outcomes in Children: A Longitudinal Database Analysis
Source: PLoS Med. 2014 Nov 4;11(11):e1001753. doi: 10.1371/journal.pmed.1001753 (PMC4219664; doi:10.1371/journal.pmed.1001753)
Supplement: Checklist S1 — STROBE research checklist. (DOCX) [file pmed.1001753.s001.docx]

**The Impact of Adenotonsillectomy on Asthma Outcomes in Children.**

**STROBE Research Checklist**

| **Title and abstract** | | |
| --- | --- | --- |
|  | 1 | (*a*) Indicate the study's design with a commonly used term in the title or the abstract  The abstract and title includes the description of the study as a longitudinal observational study of data sampled from a clinical database. |
|  |  | (*b*) Provide in the abstract an informative and balanced summary of what was done and what was found  Already Done. |
| **Introduction** | | |
| Background/rationale | 2 | Explain the scientific background and rationale for the investigation being reported  Paragraph 1, 2, 3 and 4 |
| Objectives | 3 | State specific objectives, including any prespecified hypotheses  Paragraph 4 |
| **Methods** | | |
| Study design | 4 | Present key elements of study design early in the paper  Paragraph 4, 6, 7, 9, 10, 11,12 |
| Setting | 5 | Describe the setting, locations, and relevant dates, including periods of recruitment, exposure, follow-up, and data collection  Paragraph 6,7 |
| Participants | 6 | (*a*) *Cohort study*?Give the eligibility criteria, and the sources and methods of selection of participants. Describe methods of follow-up*Case-control study*?Give the eligibility criteria, and the sources and methods of case ascertainment and control selection. Give the rationale for the choice of cases and controls*Cross sectional study*?Give the eligibility criteria, and the sources and methods of selection of participants |
|  |  | (*b*) *Cohort study*?For matched studies, give matching criteria and number of exposed and unexposed*Case-control study*?For matched studies, give matching criteria and the number of controls per case  Paragraph 7,8 |
| Variables | 7 | Clearly define all outcomes, exposures, predictors, potential confounders, and effect modifiers. Give diagnostic criteria, if applicable  Paragraph 10, 11 and 12. Supplemental Tables S1 and S2 |
| Data sources/ measurement | 8* | For each variable of interest, give sources of data and details of methods of assessment (measurement). Describe comparability of assessment methods if there is more than one group  Paragraph 6 |
| Bias | 9 | Describe any efforts to address potential sources of bias  N/A. Elements of Bias inhertent in the database were discussed in the discussion section.  Paragraph 27 |
| Study size | 10 | Explain how the study size was arrived at  Based largely on exclusion.  Paragraph 7,8 and 9 and FIGURE 1 |
| Quantitative variables | 11 | Explain how quantitative variables were handled in the analyses. If applicable, describe which groupings were chosen and why  Paragraph 13, 14 |
| Statistical methods | 12 | (*a*) Describe all statistical methods, including those used to control for confounding  Paragraph 13,14 |
|  |  | (*b*) Describe any methods used to examine subgroups and interactions  N/A |
|  |  | (*c*) Explain how missing data were addressed  N/A |
|  |  | (*d*) *Cohort study*?If applicable, explain how loss to follow-up was addressed*Case-control study*?If applicable, explain how matching of cases and controls was addressed Paragraph 7 FIGURE 1  *Cross sectional study*?If applicable, describe analytical methods taking account of sampling strategy |
|  |  | (*e*) Describe any sensitivity analyses |
| **Results** | | |
| Participants | 13* | (*a*) Report numbers of individuals at each stage of study?eg numbers potentially eligible, examined for eligibility, confirmed eligible, included in the study, completing follow-up, and analysed Paragraph 15 FIGURE 1 |
|  |  | (*b*) Give reasons for non-participation at each stage N/A (Due to database nature, all patients were participants) |
|  |  | (*c*) Consider use of a flow diagram  FIGURE 1 |
| Descriptive data | 14* | (*a*)Give characteristics of study participants (eg demographic, clinical, social) and information on exposures and potential confounders  Paragraph 15 TABLE 1, Supplemental Table S3 |
|  |  | (*b*) Indicate number of participants with missing data for each variable of interest  N/A |
|  |  | (*c*) *Cohort study*?Summarise follow-up time (eg average and total amount)  Paragraph 9, FIGURE 1 |
| Outcome data | 15* | *Cohort study*?Report numbers of outcome events or summary measures over time |
|  |  | *Case-control study?*Report numbers in each exposure category, or summary measures of exposure  Paragraph 10,11,12 FIGURE 1, TABLE 1 |
|  |  | *Cross sectional study?*Report numbers of outcome events or summary measures |
| Main results | 16 | (*a*) Report the numbers of individuals at each stage of the study?eg numbers potentially eligible, examined for eligibility, confirmed eligible, included in the study, completing follow-up, and analysed  Paragraph 15, FIGURE 1, TABLE 1 |
|  |  | (*b*) Give reasons for non-participation at each stage  N/A |
|  |  | (*c*) Consider use of a flow diagram  FIGURE 1 |
| Other analyses | 17 | Report other analyses done?eg analyses of subgroups and interactions, and sensitivity analyses  N/A |
| **Discussion** | | |
| Key results | 18 | Summarise key results with reference to study objectives  Paragraph 28.29 |
| Limitations | 19 | Discuss limitations of the study, taking into account sources of potential bias or imprecision. Discuss both direction and magnitude of any potential bias  Paragraph 23,24, 27 |
| Interpretation | 20 | Give a cautious overall interpretation of results considering objectives, limitations, multiplicity of analyses, results from similar studies, and other relevant evidence  Paragraph 28.29 |
| Generalisability | 21 | Discuss the generalisability (external validity) of the study results  Paragraph 28.29 |
| **Other information** | | |
| Funding | 22 | Give the source of funding and the role of the funders for the present study and, if applicable, for the original study on which the present article is based  Funding was disclosed in the online submission |
