# Supplementary figures and images for: Association of Adenotonsillectomy with Asthma Outcomes in Children: A Longitudinal Database Analysis
Source: PLoS Med. 2014 Nov 4;11(11):e1001753. doi: 10.1371/journal.pmed.1001753 (PMC4219664; doi:10.1371/journal.pmed.1001753)

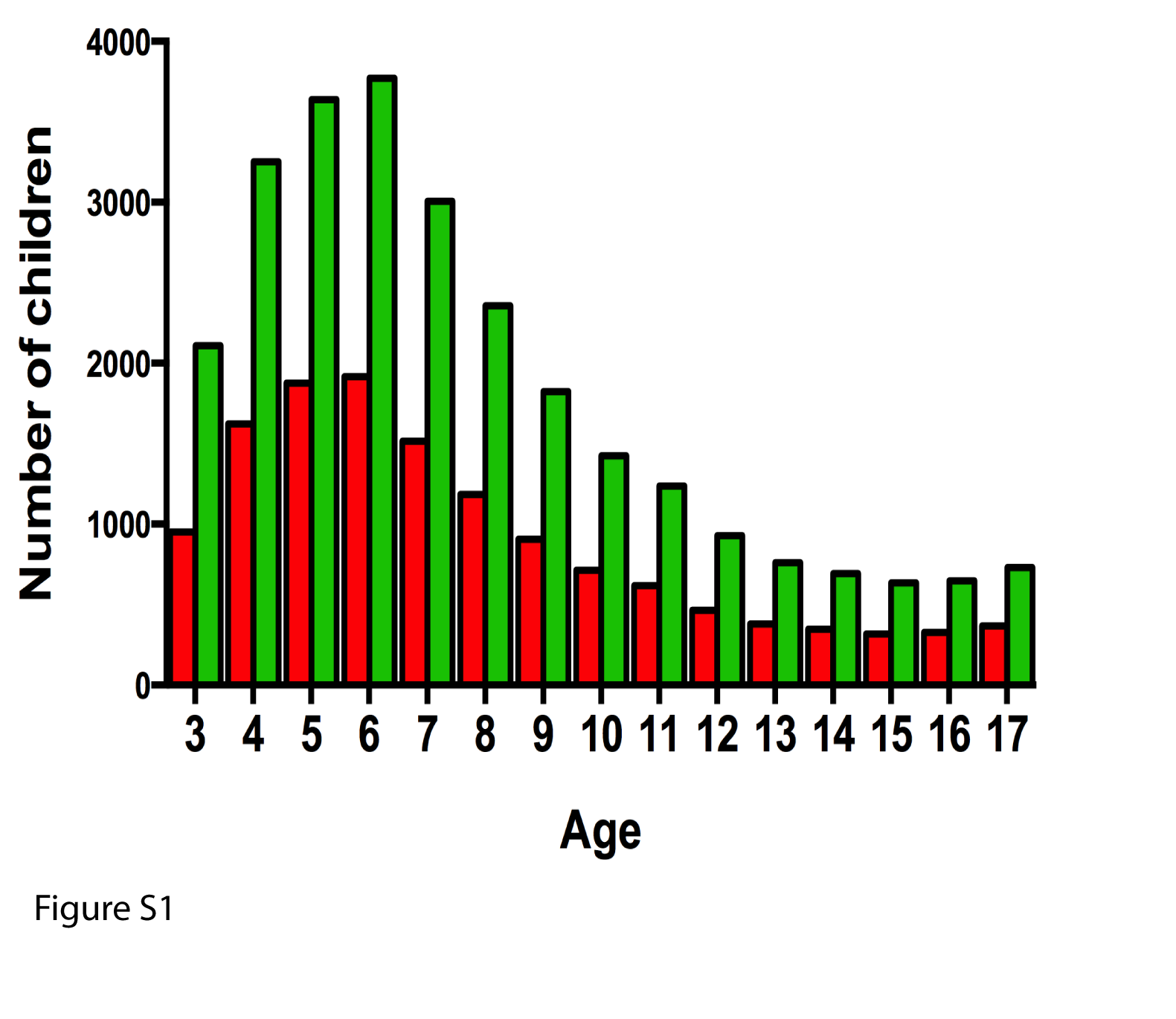

Supplement: Figure S1 — Age distribution of the AT+ and AT− groups. Matched AT− group children were chosen using a 2∶1 ratio; hence, at each age point there is an anticipated doubling in the AT− group (green bars) for the corresponding AT+ group (red bars). (TIF) [file pmed.1001753.s002.tif]
