# Supplementary material for: Association of Adenotonsillectomy with Asthma Outcomes in Children: A Longitudinal Database Analysis
Source: PLoS Med. 2014 Nov 4;11(11):e1001753. doi: 10.1371/journal.pmed.1001753 (PMC4219664; doi:10.1371/journal.pmed.1001753)
Supplement: Table S1 — ICD-9-CM and CPT codes used as study outcomes. (DOCX) [file pmed.1001753.s003.docx]

Supplemental Table S1 – ICD-9 and CPT Codes used as Study Outcomes

| **Item** | **Codes** | **(+) AT** | | **(-) AT** | |  | **(+) AT** | **(-) AT** |
| --- | --- | --- | --- | --- | --- | --- | --- | --- |
|  |  | 1 year Pre | 1 year Post | 1 year Pre | 1 year Post | p Value | % Reduction | % Reduction |
|  |  |  |  |  |  |  |  |  |
| Extrinsic Asthma with Acute Exacerbation | 493.02 | 605 | 426 | 861 | 849 | p<0.0001 | 30.0% (20.5-37.7) | 1.4% (-8.3-10.2) |
| Intrinsic Asthma with Acute Exacerbation | 493.12 | 127 | 73 | 154 | 137 | p=0.0206 | 42.5% (23.4-56.9) | 11.0% (-11.9-29.3) |
| Chronic Obstructive Asthma with Acute Exacerbation | 493.22 | 21 | 21 | 42 | 39 | p=0.8518 | 0.0% (-0.83-0.45) | 7.1% (-43.5-39.9) |
| Asthma unspecified with Acute Exacerbation | 493.92 | 1490 | 1046 | 2346 | 2311 | p<0.0001 | 29.8% (24.3-34.9) | 1.5% (-4.1-6.8) |
| **SUM** |  | 2243 | 1566 | 3403 | 3336 | p<0.0001 | 30.2% (25.9-34.3) | 2.0% (-2.5-6.3) |
|  |  |  |  |  |  |  |  |  |
| Extrinsic Asthma with Status Asthmaticus | 493.01 | 145 | 104 | 226 | 215 | p=0.0808 | 28.3% (7.8-44.2) | 4.9% (-14.6-21.0) |
| Intrinsic Asthma with Status Asthmaticus | 493.11 | 26 | 15 | 20 | 37 | p=0.0076 | 42.3% (-8.9-69.4) | -85.0%(-218.6--7.4) |
| Chronic Obstructive Asthma with Status Asthmaticus | 493.21 | 15 | 6 | 10 | 9 | p=0.3284 | 60.0% (-3.1-84.5) | 10.0% (-121.5-63.4) |
| Asthma unspecified with Status Asthmaticus | 493.91 | 376 | 224 | 581 | 517 | p<0.0001 | 40.4% (29.8-49.4) | 11.0% (-0.1-20.9) |
| **SUM** |  | 562 | 349 | 837 | 778 | p<0.0001 | 37.9 % (29.2-45.6) | 6.8% (-2.6-15.4) |
|  |  |  |  |  |  |  |  |  |
| Acute Bronchospasm | 519.11 | 251 | 188 | 448 | 431 | p=0.04 | 25.1% (0.7-37.9) | 3.8% (-9.7-15/6) |
| Wheezing | 786.07 | 839 | 501 | 1222 | 1261 | p<0.0001 | 40.3% (33.5-46.4) | -3.2% (-11.4-4.4) |
| Spirometry | 940.1 | 1615 | 1398 | 1820 | 2471 | p<0.0001 | 13.4% (7.4-19.1) | -35.8% (-43.9--28.1) |
| Continuous Inhalation for First Hour | 946.44 | 2225 | 1563 | 3054 | 3251 | p<0.0001 | 30.0%(25.4-33.8) | -6.5% (-11.5--1.6) |
| Intubation | 96.04 | 22 | 16 | 49 | 29 | p=1.00 | 27.3% (-38.44-61.8) | 40.8% (6.4-62.6) |

Supporting Information
