# Supplementary material for: Association of Adenotonsillectomy with Asthma Outcomes in Children: A Longitudinal Database Analysis
Source: PLoS Med. 2014 Nov 4;11(11):e1001753. doi: 10.1371/journal.pmed.1001753 (PMC4219664; doi:10.1371/journal.pmed.1001753)
Supplement: Table S2 — Asthma pharmacy classes used as study outcomes. (DOCX) [file pmed.1001753.s004.docx]

Supplemental Table S2– Asthma Pharmacy Classes Used as Study Outcomes

| **Drug Classes** | |
| --- | --- |
| **Bronchodilator (BD)** | |
|  | Albuterol |
|  | Levalbuterol |
|  | Ipratropium |
| **Inhaled Corticosteroid (ICS)** | |
|  | Fluticasone Furorate |
|  | Fluticasone Propionate |
|  | Fluticasone Propionate, Micronized |
|  | Budesonide |
|  | Budesonide, Micronized |
|  | Mometasone Furoate |
|  | Beclomethasone Dipropionate |
|  | Beclomethasone Dipropionate Monohydrate |
|  | Beclomethasone Dipropionate Micronized |
|  | Ciclesonide |
| **Inhaled Corticosteroid and Long Acting Beta Agonist (ICS/LABA)** | |
|  | Fluticasone Propionate /Salmeterol Xinafoate |
|  | Budesonide/Formoterol Fumarate |
|  | Mometasone Furoate /Formoterol Fumarate |
| **Leukotriene Receptor Antagonists (LTRA)** | |
|  | Montelukast Sodium |

Supporting Information
