# Supplementary material for: Association of Adenotonsillectomy with Asthma Outcomes in Children: A Longitudinal Database Analysis
Source: PLoS Med. 2014 Nov 4;11(11):e1001753. doi: 10.1371/journal.pmed.1001753 (PMC4219664; doi:10.1371/journal.pmed.1001753)
Supplement: Table S3 — Demographic summary of children included in and excluded from the AT+ group. (DOCX) [file pmed.1001753.s005.docx]

|  | | | **(+) AT** | **(+) AT** | **p value** |
| --- | --- | --- | --- | --- | --- |
|  |  |  | **Included** | **Excluded** |  |
| **Number** | | | 13506 | 38288 |  |
| **Diagnostic Code** | | |  |  |  |
| **474.1x** | | Hypertrophy of tonsils and adenoids | 5942 (44%) | 11426 (30%) | <0.0001 |
| **474.0x** | | Chronic tonsillitis and adenoiditis | 3062 (23%) | 2894 (8%) |  |
| **474.1x & 474.0x** | | Hypertrophy of tonsils and adenoids & Chronic tonsillitis and adenoiditis | 2344 (17%) | 23776 (62%) |  |
| **327.20,327.23,327.24,327.26,327.29,780.51,780.53,780.57,786.03,780.50, 780.56,780.59** | | Sleep Apnea, Snoring and/or Sleep Disturbance | 3603 (27%) | 10707 (28%) |  |
| **Age** | | | 7.70±3.65 | 7.2±3.83 | <0.0001 |
| **Gender** | | |  |  |  |
|  | Male | | 7440 (55%) | 21027 (54.9%) | 0.7424 |
|  | Females | | 6066 (45%) | 17261 (45.1%) |  |
| **Available Pharmacy Reimbursement Claims** | | | 10,663 (79%) | 31765 (83%) | 0.4709 |
| **Geographical Location** | | |  |  |  |
|  | METROPOLITAN COUNTIES | |  |  |  |
|  | Counties of metro areas of 1 million population or more | | 6116 (45.2%) | 18113 (47.7%) | 1.00 |
|  | Counties in metro areas of 250,000 ‑ 1,000,000 population | | 2746 (20.3%) | 7182 (18.9%) |  |
|  | Counties in metro areas of fewer than 250,000 population | | 1896 (14.0%) | 4946 (13.0%) |  |
|  | NONMETROPOLITAN COUNTIES | |  |  |  |
|  | Urban population of 20,000 or more, adjacent to a metro area | | 756 (5.6%) | 2177 (5.7%) |  |
|  | Urban population of 20,000 or more, not adjacent to a metro area | | 278 (2.1%) | 859 (2.3%) |  |
|  | Urban population of 2,500‑19,999, adjacent to a metro area | | 1024 (7.6%) | 2631 (6.9%) |  |
|  | Urban population of 2,500‑19,999, not adjacent to a metro area | | 442 (3.3%) | 1381 (3.6%) |  |
|  | Completely rural or less than 2,500 urban population, adjacent to a metro area | | 124 (0.9%) | 360 (1.0%) |  |
|  | Completely rural or less than 2,500 urban population, not adjacent to a metro area | | 124 (0.9%) | 324 (0.9%) |  |
