# Supplementary material for: Association of Adenotonsillectomy with Asthma Outcomes in Children: A Longitudinal Database Analysis
Source: PLoS Med. 2014 Nov 4;11(11):e1001753. doi: 10.1371/journal.pmed.1001753 (PMC4219664; doi:10.1371/journal.pmed.1001753)
Supplement: Table S4 — CPT codes used for sleep apnea diagnostic testing. (DOCX) [file pmed.1001753.s006.docx]

Supplemental Table S4– CPT Codes used for Sleep Apnea Diagnostic Testing

| Diagnostic Test | CPT Code |
| --- | --- |
| Sleep study, simultaneous recording of, heart rate, oxygen saturation, respiratory airflow, and respiratory effort, Unattended | 958.06 |
| Sleep study, simultaneous recording of ventilation, respiratory effort, ECG or heart rate, and oxygen saturation, Attended | 958.07 |
| Polysomnography; sleep staging with 1-3 additional parameters of sleep, Attended | 958.08 |
| Polysomnography; sleep staging with 4 or more additional parameters of sleep, Attended | 958.10 |
| Polysomnography; sleep staging with 4 or more additional parameters of sleep, with initiation of continuous positive airway pressure therapy or bilevel ventilation, Attended | 958.11 |
| Overnight Pulse Oximetry | 94762 |
| Home Sleep Study Test with Type II Portable Monitor, Unattended | G0398 |
| Home Sleep Study Test with Type III Portable Monitor, Unattended | G0399 |
| Home Sleep Study Test with Type IV Portable Monitor, Unattended | G0400 |

Supporting Information
